# Community health workers serving Veterans with chronic obstructive pulmonary disease: a pilot intervention development and feasibility study

**DOI:** 10.1186/s40814-025-01711-8

**Published:** 2026-01-03

**Authors:** C. Bradley Kramer, Mayuree Rao, David B. Coultas, Jessica Young, George Sayre, Emily R. Locke, Tiffanie Fennell, Bryan J. Weiner, Karin M. Nelson, Jessica Jones-Smith, Vincent S. Fan

**Affiliations:** 1 VA Puget Sound Health Care System, Seattle-Denver Center of Innovation for Veteran-Centered and Value-Driven Care, Seattle, WA USA; 2 University of Washington, School of Public Health, Department of Health Systems and Population Health, Seattle, WA USA; 3 Public Health–Seattle & King County, Chronic Disease and Injury Prevention Section, Seattle, WA USA; 4 VA Puget Sound Healthcare System, General Medicine Service, Seattle, WA USA; 5 University of Washington, School of Medicine, Department of Medicine, Seattle, WA USA; 6 VA Portland Healthcare System, Division of Hospital and Specialty Medicine, Portland, OR USA; 7 Oregon Health & Science University, Department of Medicine, Portland, OR USA; 8 University of Washington, School of Medicine, Department of Psychiatry and Behavioral Sciences, Seattle, WA USA; 9 University of Washington, School of Public Health, Department of Global Health, Seattle, WA USA

**Keywords:** Chronic obstructive pulmonary disease, Community health worker, Peer support, Qualitative

## Abstract

**Background:**

Chronic obstructive pulmonary disease (COPD) causes significant morbidity and mortality and is a substantial burden on healthcare systems. Disease self-management programs can reduce symptoms, lower hospitalizations, and improve patient quality of life. We adapted and piloted a COPD self-management program delivered by community health workers (CHWs) to Veterans. This study aimed to assess participants’ perceived acceptability, appropriateness, and feasibility of the intervention. We investigated barriers and facilitators to achieving disease self-management practices. We explored participants’ COPD health outcomes. Finally, we gathered insights from participants and CHWs to inform potential improvements.

**Methods:**

Nine Veterans enrolled in the 12-week intervention and received a series of 9 CHW home, phone, or video visits. We assessed perceived intervention acceptability, appropriateness, and feasibility qualitatively and quantitatively. We conducted a qualitative content analysis of semi-structured interviews with intervention participants and their CHWs on overall perceptions of the intervention. An additional analysis phase included translation of the results into suggestions for future adaptations by the multi-disciplinary investigator team. We administered surveys on self-reported acceptability, appropriateness, and feasibility of the intervention, as well as COPD health outcomes.

**Results:**

The intervention had high participant-perceived acceptability (4.2 ± 0.8), appropriateness (4.3 ± 0.5), and feasibility (4.2 ± 0.6), on a scale from 1 to 5. Interviewed participants highlighted the benefits of CHW-led education on COPD understanding, breathing techniques, and proper inhaler use. Participants further emphasized the social support and connection to resources provided by the program. Overall, the participants and their CHW providers shared feedback that demonstrate the acceptability, appropriateness, and feasibility of this intervention. Exploratory results also showed improved health-related outcomes. Some suggested adaptations emerged, such as including optional caregiver involvement and addressing potential stigma related to COPD.

**Conclusions:**

The pilot presents a promising CHW-led intervention to improve COPD self-management. These initial results suggest the intervention is acceptable, feasible, and appropriate and could improve health outcomes, including quality of life. Future programs or randomized controlled trial design could benefit from the study’s recommended adaptations.

**Supplementary Information:**

The online version contains supplementary material available at 10.1186/s40814-025-01711-8.

## Key messages regarding feasibility

- The CHW model has proven effective in many chronic diseases, but the model has not been established for COPD.
- This study suggests a CHW intervention was acceptable, appropriate, and feasible for Veterans with COPD.
- These primarily qualitative results present patient-centered feedback to help improve CHW COPD interventions for Veterans and others.

**Supplementary Information:**

The online version contains supplementary material available at 10.1186/s40814-025-01711-8.

## Background

Chronic obstructive pulmonary disease (COPD) affects more than 10% of US adults over 40 years of age [1] and 14–19% of Veterans [2–4]. The disease results in high hospital admission, readmission, and mortality rates, contributing to over $37 billion annually borne by the US health care system [1, 5]. The burden of COPD can be reduced through self-management practices, including medication adherence, proper inhaler technique, and routine physical activity [6]. To improve symptoms and reduce hospitalizations, COPD self-management programs staffed by health care professionals, such as nurses or respiratory therapists, have shown improvements in health-related quality of life and reductions in hospitalizations [6–8]. However, there are several barriers to implementation of self-management programs in clinical practice including complex social, emotional, and medical needs for patients and insufficient time or skills for health care providers [9].

Community health worker (CHW) programs that address chronic disease are common and effective, with increasing evidence for diabetes, hypertension, cancer screening, and asthma [10–13]. These programs improve participant health through disease self-management education and behavior change support. However, a systematic review in 2019 found no published studies of CHW programs addressing COPD [14]. Two subsequent COPD interventions including peers and lay health workers (allied workforces, sometimes classified as CHWs [15]) had favorable results in improving self-efficacy [16] and medication adherence [17], with an editorial call for more research in this area [18].

Concurrent with the development of these recent studies, we piloted a novel COPD self-management intervention delivered by CHWs in the participant’s home. This intervention served Veterans receiving primary care at the VA, a population that experiences disproportionately high rates of COPD and associated morbidity [4, 19, 20]. The intervention was adapted from a program previously tested in a clinical setting using health coaches to deliver self-management education and support [21, 22].

As a primary study aim, we examined three early-phase implementation outcomes of the pilot intervention (acceptability, appropriateness, and feasibility). As a secondary study aim, we examined health outcomes to inform intervention refinement and the design of a fully scaled randomized controlled trial to address COPD outcomes disparities among Veterans. We also sought to reveal broader challenges and opportunities for CHW programs to address COPD.

## Methods

All study procedures were approved by VA (#1588037) and UW (#00007561) Institutional Review Boards. All participants (Veterans and CHWs) completed informed consent. For reporting, we followed the Consolidated Criteria for Reporting Qualitative Studies (COREQ) guidelines (Additional File 1) [23]. We retrospectively registered the study with clnicaltrails.gov (NCT06350799).

### Setting and participants

**Fig. 1:**
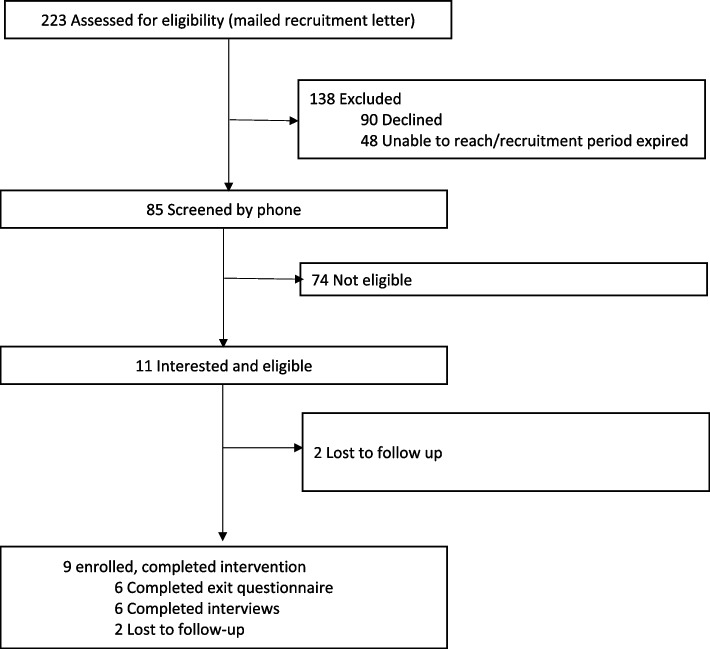
Flow diagram of study participant screening, eligibility, enrollment, and completion

Patients receiving care at the VA Puget Sound Health Care System were recruited for this study. We used a VA administrative database to identify patients with a visit with an ICD-10 code for COPD in the past year. Eligible patients were enrolled in primary care, diagnosed with COPD, age > 45 years, English speaking, clinically stable for at least 1 month prior to enrollment, and had access to the internet to participate in telehealth appointments. In order to target patients at high risk for poor outcomes, we further limited eligibility to those with ≥ 1 exacerbation in the past year treated with prednisone and/or antibiotics, an emergency department visit, or a hospital admission. We sent recruitment letters to 223 potentially eligible Veterans. Of the 175 successfully contacted, 11 were interested and eligible, and 9 were enrolled (Fig. 1).

### CHW background and training

Three CHWs participated in the pilot intervention. The CHWs worked at the local public health department, with prior work in a clinical program focused on asthma and experience participating in clinical trials [24, 25]. The CHWs serve the greater King County area, and recruitment was limited to this region. They are not Veterans themselves, but two have Veterans in their families. They received 40 h of training through sessions with the study clinicians (VF and DC) and online courses through the American Lung Association. They met routinely with the study staff and principal investigator (VF) to review all visits and had access to on-call support if needed. They also received technical assistance for video conferencing with study staff.

### Sample size

We planned to enroll up to ten participants into the study, which would provide sufficient data capture for qualitative analysis [26]. This study did not have dedicated funding, which limited the amount of staffing available. We sent out small batches of recruitment letters, reassessing availability of CHW and recruitment staff for study continuation after processing each batch.

### Intervention

The COPD-CHW Home Visits study was initially designed to be delivered through a combination of home and virtual visits (video or phone), as described below and in the TIDieR checklist (Additional File 3). The intervention became fully virtual after the second participant was enrolled due to the COVID-19 pandemic. Per patient preference, CHW visits were conducted either through video conference technology (VA Video Connect) or by phone, with the exception of the first two participants that were offered home visits. Participants were offered additional phone calls between visits to check-in on their COPD self-management goals and confirm their upcoming appointments.

Veteran participants were scheduled for 9 CHW visits over 12 weeks. As part of the first visits, the CHW performed a needs assessment that included a checklist-based home environment review, which could be done by video or phone when home visits were not possible during the COVID-19 pandemic period. The CHW learned about the Veteran’s priorities for their COPD, which they incorporated into a COPD management plan. Using motivational interviewing techniques, the CHW discussed any challenges that might exist to implementing the plan and approaches to address those concerns. The CHW worked with the Veteran to set a schedule to cover the 9 visits on a near-weekly basis. We utilized materials from the COPD-Self-Management Activation Research Trial (COPD-SMART) intervention [22], which was a 9-month intervention with COPD education and 25 physical activity sessions. The participant received the COPD-SMART manuals to use for visits, but the CHWs only covered the introductory materials focused on COPD self-management (6 sessions) and developing a physical activity plan (3 sessions). The COPD self-management sessions included (a) understanding COPD and its impact; (b) communicating with health care providers; (c) understanding medications; (d) non-pharmacologic strategies for controlling symptoms; (e) exacerbation action plans; (f) enhancing physical activity; (g) eating healthy; (h) smoking cessation; and (i) mood. To enhance the section on understanding medications, the CHW trained the Veteran on proper use of an inhaler, using the Teach-to-Goal method [27, 28]. The final three sessions covered motivation for physical activity: getting ready for healthy behavior change, and making a plan to overcome barriers and achieve benefits.

### Measures

This study used a multiple method, descriptive approach to describe the pilot study results. Veteran participants completed enrollment and exit questionnaires (Questionnaire contents are presented in Additional File 4). Exit questionnaires were sent for completion 4 months after enrollment, about 1 month after intervention completion. The participants and CHWs were invited to participate in a 30–45 min qualitative interview with study staff. Participants were interviewed from June 2020 to January 2021. CHWs were interviewed in April 2023. Participation in all components was voluntary. No monetary incentive was provided.

### Qualitative data collection—participant and CHW interviews

Interviewers and primary analysts were a doctoral student (CBK) and a physician researcher (MR) under the supervision of a qualitative methods expert (GS). Interviewers made up to three attempts to reach all the Veteran participants and CHWs by phone to schedule an interview time. All interviews were recorded and transcribed. We sought to receive as much feedback from pilot participants as possible, rather than setting an a priori limit or seeking data saturation [26].

The semi-structured interview guides included questions on overall perceptions of the intervention components, interactions with CHWs/Veterans, and suggestions for future implementation (Additional File 2). We sought to elicit barriers and facilitators. The interview guides and analysis were informed by Social Cognitive Theory [29], and the conceptual model presented by Postma et al. [11], referencing how common behavior change frameworks function in the design of inter-personal and multi-faceted CHW interventions. The theoretical domains framework (TDF) was used in the analysis to examine the individual and interpersonal components of the intervention [30]. As a meta-framework, the TDF synthesizes 33 theories of behavior change into 14 domains. Thus, the results defined through the TDF domains can be compared across other theory-informed models and interventions, to better understand the mechanisms of behavior change and how they support intervention activities. Finally, we apply these to the Proctor et al. implementation outcomes [31], which contextualizes how acceptability, feasibility, and appropriateness fit into the phases of effective intervention development.

### Quantitative data collection—participant questionnaires

#### Intervention acceptability, appropriateness, and feasibility

The exit questionnaire included standardized measures of intervention acceptability (satisfaction with various aspects of the intervention, e.g., content, complexity, comfort, delivery, and credibility), appropriateness (perceived fit, relevance, or compatibility of the intervention for the Veteran, the VA, and to address COPD), and feasibility (the extent to which the intervention can be successfully used or carried out in the patient’s home and supported by the VA) [32]. Each of these three outcomes had four prompts rated on a 5-point scale (completely disagree, disagree, neither agree nor disagree, agree, and completely agree) with 5 representing “completely agree” [32]. The questionnaires also included general questions about satisfaction with the intervention and its components.

#### Health outcomes

Several measures were selected because they are standard outcomes for COPD trials including quality of life, respiratory symptoms, and psychological symptoms. Measures assessing the key health behaviors of inhaler adherence and physical activity were also included. The enrollment and exit questionnaires were completed by mail with additional phone and video assistance if necessary. The 20-item Chronic Respiratory Disease Questionnaire (CRQ) measured health status and has 4 subscales (dyspnea, fatigue, emotional function and mastery) with a range of 1–7 with higher scores indicating better quality of life. A change of 0.5 is considered clinically meaningful and designed for assessment of change in health-related quality of life [33–35]; COPD Assessment Test (CAT) measuring respiratory symptoms and impact of the disease over time with a score of 10 or greater considered a high burden of symptoms [36–38]; psychological symptoms were measured with the Hospital Anxiety and Depression Score (HADS) with two scales to measure anxiety and depression symptoms with a cut-off of ≥ 8 or ≥ 11 are used to identify those with possible depression [39–41]; medication adherence was measured with the Adherence to Refills and Medications Scale (ARMS) which has a range between 7 and 28, with a score of 7 considered to indicate adherence and scores higher than 7 considered to indicate some degree of nonadherence [42, 43]; and Physical Activity Scale for the Elderly (PASE), a validated questionnaire of leisure, occupational, and household activities (range 0 to 400; higher scores indicate greater physical activity) [44, 45].

### Analysis

#### Qualitative data analysis

We analyzed transcripts using simultaneous deductive and inductive content analysis [46, 47]. Deductive content analysis is more structured and consists of identifying meaningful units that fit within a priori categories. Inductive content analysis consists of open/unstructured coding and allows for the identification of emergent or unexpected themes. The a priori categories for deductive analysis were informed by the project’s proposed conceptual model and TDF framework. We reviewed transcripts for quality assurance and uploaded into ATLAS.ti v.9 [48] for analysis. One researcher (BK) reviewed all interviews multiple times, applied codes, and prepared preliminary results for review. Additional authors iteratively reviewed summarized results and partial transcripts through a series of team meetings and presentations, contributing to the analysis and interpretation, providing a multi-disciplinary perspective (MR, DBC, GS, JY, EL, BJW, VF). The authors represent practicing clinicians (primary care, pulmonology, behavioral health) who serve Veterans, public health practitioners, and career researchers. Iterative review encouraged active engagement and reflexivity of positionality.

We conducted three phases of qualitative analysis following methods on adapting interventions based on qualitative results [49]. Phase 1 had the primary goal of analyzing the qualitative and quantitative data from participants. We analyzed participant interviews. We consolidated deductive and emergent themes through the qualitative methods described above. We sorted the themes by categories. For themes related to health behavior change, the TDF can assist in the translation of the themes into intervention modifications, addressed in the next phase[30]. Phase 2 had the primary goal of identifying potential intervention components that could be improved for a future study. The investigators developed recommended adaptations based on interpretation of participant feedback. We included some of the suggested adaptations in the CHW interviews for additional feedback and validation. Phase 3 had the primary goal of adding the CHW perspective to the final results. We analyzed the CHW interviews through the qualitative methods described above. We then compared the results to the participant interview results, adding the CHW perspective. In addition, we included analysis of CHW insights on the recommended intervention adaptations from phase 2.

#### Quantitative data analysis

We present summary survey results analyzed using STATA version 18 [50]. Descriptive statistics were reported via means and standard deviations. Differences between baseline and final measurements were evaluated through a two-sided, paired *t* test. Given the small sample size, the analysis is exploratory and intended to inform future work, not to formally test effectiveness.

## Results

Participants enrolled in the study between September 2019 and June 2020. We identified 223 potentially eligible Veterans with COPD and screened 85 for full study criteria by telephone (Fig. 1). We successfully enrolled nine out of the eleven eligible participants (81%), and 100% completed the nine planned study visits. The final Veteran participant data included exit questionnaires (*n* = 6) and follow-up interviews (*n* = 7), with incomplete overlap (*n* = 5) between these two data sources. All participants who completed the baseline and exit questionnaires were male, had a mean age of 73 years, and five (83%) identified as white, non-Hispanic. Additional income, education, insurance states, and living situations are described in Table 1.

**Table 1:** Participant characteristics

Variables	Completed study visits *N* = 9	Completed final survey *N* = 6
Characteristics		
Age, mean (SD)	67.8 (10.5)	72.7 (7.5)
Sex (Male), *n* (%)	9 (100.0)	6 (100.0)
Weight, pounds, mean (SD)	210 (30.4)	219.6 (36.7)
Height, inches, mean (SD)	70.3 (2.5)	71.3 (2.3)
Married/significant other, *n* (%)	5 (55.5)	3 (50.0)
Live alone, *n* (%)	3 (33.3)	2 (33.3)
Race, White, *n* (%)	7 (77.8)	5 (83.3)
Education, *n* (%)		
Some College	6 (66.7)	4 (66.7)
College Grad	3 (33.3)	2 (33.3)
Employed, *n* (%)		
Full-time and part-time	4 (44.4)	2 (33.3)
Retired and Not working	5 (55.5)	4 (66.7)
Income, *n* (%)		
< $30,000/year	3 (33.3)	1 (16.6)
$30,000-$60,000/year	4 (44.4)	2 (33.3)
> $60,000/year	4 (44.4)	3 (50.0)
Insurance Info, *n* (%)		
Medicare	8 (88.9)	5 (83.3)
Medicaid	1 (11.1)	0 (0.00)
Tricare or Private	4 (44.4)	2 (33.3)
Copay is a burden, *n* (%)		
Always or sometime	5 (55.5)	2 (33.3)
No	4 (44.4)	4 (66.7)
Difficult to pay bills, *n* (%)		
Very or somewhat difficult	2 (25.0)	1 (16.6)
Not too or not at all difficult	6 (75.0)	5 (83.3)

### Acceptability, appropriateness, and feasibility

#### Quantitative—participant questionnaires

The results of the acceptability (mean 4.2, SD 0.8), appropriateness (4.3, SD 0.5), and feasibility (4.2, SD 0.6) assessments were all positive, between agree (4) and strongly agree (5) (Table 2).

**Table 2:** Pre-/post-questionnaire results for 9-week pilot CHW study (*N* = 6)

	Baseline mean (SD)	Final mean (SD)	Difference, mean change (95% CI)
CAT score	16.0 (5.8)	12.0 (6.6)	− 4 (− 7.51 to − 0.49)
CRQ scores			
Dyspnea	4.8 (1.3)	5.5 (0.9)	0.7 (− 0.12 to 1.52)
Fatigue	3.5 (1.0)	4.6 (0.8)	1.1 (0.11 to 2.06)
Emotional	5.1 (1.1)	5.9 (0.7)	0.8 (0.18 to 1.44)
Mastery	5.2 (1.3)	6.1 (0.9)	0.9 (− 0.09 to 1.92)
HADS-scores			
Anxiety	4.3 (3.3)	3.8 (2.2)	− 0.5 (− 2.96 to 1.96)
Depression	5.3 (2.6)	5.5 (2.9)	0.2 (− 2.8 to 3.09)
ARMS score	9.5 (5.2)	7.7 (3.9)	− 1.8 (− 1.09 to 4.75)
PASE Score	200.3 (113.3)	132.2 (74.2)	− 68.1 (− 117.97 to − 18.12)
Acceptability, appropriateness, and feasibility			
Acceptability (AIM) Score	–	4.2 (0.8)	–
The COPD Home Visits program meets my approval	–	4.3 (0.8)	–
The COPD Home Visits program is appealing to me	–	4.2 (1.0)	–
I like the COPD Home Visits program	–	4.2 (1.0)	–
I welcome the COPD Home Visits program	–	4.2 (1.0)	–
Appropriateness (IAM) Score	–	4.3 (0.5)	–
The COPD Home Visits program seems fitting	–	4.3 (0.5)	–
The COPD Home Visits program seems suitable	–	4.3 (0.5)	–
The COPD Home Visits program seems applicable	–	4.3 (0.5)	–
The COPD Home Visits program seems like a good match	–	4.0 (0.9)	–
Feasibility (FIM) Score	–	4.2 (0.6)	–
The COPD Home Visits program seems workable	–	4.2 (0.8)	–
The COPD Home Visits program seems possible	–	4.2 (0.8)	–
The COPD Home Visits program seems doable	–	4.3 (0.5)	–
The COPD Home Visits program seems easy to use	–	4.2 (0.8)	–

*SD* standard deviation, *CI* confidence interval, *CAT* COPD assessment test (↑ scores worse), *CRQ* Chronic Respiratory Questionnaire (↑ scores better), *HAD* Hospital Anxiety and Depression (↑ scores worse), *ARMS* Adherence to Refills and Medications Scale (↑ scores worse), *PASE* physical activity scale for the elderly (↑ scores better), *AIM* acceptability of intervention measure (↑ scores better), *IAM* intervention appropriateness measure (↑ scores better), *FIM* feasibility of intervention measure (↑ scores better)

Participants responded in the survey to questions on satisfaction with elements of the interventions, asked as yes/no or non-standardized formats. Surveyed participants were overall satisfied with the program, 100% agreeing or strongly agreeing that they would recommend it to a friend. All surveyed Veterans (*n* = 6) thought the number of visits was “just right.” All surveyed participants preferred face-to-face visits, with two-thirds ranking home visits highest in modality preference, and one-third ranking video visits highest.

#### Qualitative phase 1: participant interviews

The participant responses are organized in three categories: intervention components, connections to additional health care and social support, and disease self-management.

### Intervention components

#### Number and cadence of visits

In the interviews, Veteran participants added, “Being selfish, I suppose there could have been more [visits]” and “I would have liked having maybe an extra week.” All participants said weekly visits worked well. A couple participants elaborated on the frequency of visits, adding that “it gave me a lot to think about within the week” and “if they called a couple times a week then that might be too much.”

#### Modality (home, video, and phone)

We asked about the feasibility of video visits, asking the few participants that shared frustrations with joining video visits to elaborate further. One responded saying that they still preferred video visits, stating “it’s more personal, it’s more interesting. So, it makes the time go.” Participants who elaborated on phone visits spoke to its appropriateness, saying it “wasn’t much different than having [video].” Another participant added that “it was easier for me to be on the phone, being on the road a lot with work.”

### Care connections

#### Connection with PCPs

Most participants did not engage with their primary care provider about the program. When asked if their primary care provider and the CHW should communicate with each other, all but one participant agreed. The participants explained that it would help for the primary care team to know about their enrollment and that both health care professionals might have helpful information from “[their] side of the shop.” Another participant thought that “the two of them talking, they might’ve come up with something that would’ve been really helpful.” The one participant that preferred the CHW not talk to their primary care team did not elaborate when asked.

#### Involvement of caregiver, family, and friends in the program

All interviewed participants replied that they would not want to have their family or friends involved in this program. They added “I do not see where that would help,” “basically my family knows about it,” and “I’d rather deal with it by myself.” One participant noted that they can see value, but it’s not necessary for them because “my family is involved, they understand.”

#### Connection to other health programs

Half of interviewed participants reported this being their first health-related care management program. For all, this was the first program focused on their respiratory disease. Three participants had previous experience with cardiac and diabetes focused programs.

### Disease self-management

Participating Veterans spoke about the program activities and health education. Responses provided insight on the barriers and facilitators to intervention impact on achieving self-management practices. Several themes emerged spanning the following TDF-domains: Knowledge; Skills; Social Influences; and Environment, Resources, and Context.

#### Knowledge: understanding COPD

All participants reported that participating in the intervention increased their knowledge and understanding of COPD. Most of the participants described program knowledge about the basics of COPD as a facilitator. One participant described being diagnosed with COPD while struggling with other diseases and “was not well enough to understand it.” This intervention “helped me understand things that I really didn’t understand.” Other participants added similar comments when describing the most useful part of the program for them: “when it told me what COPD was” and “education of what COPD actually is and all of that.”

In coming to terms with the disease, one participant perceived that potential stigma, due to common knowledge about the harms of cigarette use, may be a barrier to participate in COPD programs for their peers:

I could see though where some people wouldn’t want to [enroll in this program], and would be apprehensive in doing it as far as talking to some stranger about something that you created yourself. I mean, it’s essentially what you’ve done. I feel kind of guilty about that. Nobody held a gun to my head and made me smoke for 50 years. I did that myself.

#### Skills: breathing techniques and proper inhaler use

Most participants discussed the breathing techniques taught by the CHW from the education materials (e.g., pursed lip breathing) as the most useful part of the program. These practices were helpful in controlling their breathing and increasing their ability to perform physical activity. They described it as “how to get my breathing under control a bit more to try to calm it down”; “how to breathe, how to get myself out of it. That was a big thing for me”; and “it taught me how to breathe to try to catch my breath. How to get my energy back.” One participant described how it allowed them to be more physically active: “by using the breathing techniques she taught me, I’m able to walk up those hills all the way now. And I think that’s great.”

A couple of participants described the benefits of learning proper inhaler techniques: “I actually hadn’t had a spacer for a really long time, and she actually helped me to get a spacer. I didn’t know that it was the more proper way to actually use the inhaler.”

#### Social influences: social support and social isolation

Several Veterans shared that they appreciated connecting with other people. One stated, “maybe just even having contact with other people is important." Others described the interaction in the context of motivation and empathy. Another participant addressed loneliness and isolation:

The Community Health Worker Program is really a good thing. There’s so many of us that are alone….And it’s nice to have somebody who understands the problems and to be able to talk to them about that… there’s so many of us that don’t have family.

#### Environment context and resources: connecting to social and community resources

The participants described CHW connections made to clinical or community resources. A few participants reported a community resources connection as a facilitator to improving their health and social needs. They reported receiving flyers and details about stress management programs. One participant was connected to Veteran resources and benefits that improved their economic situation. Another described support from the CHW in engaging with their employer about their COPD. None of the participants received connections to clinical resources.

### Qualitative phase 2: intervention adaptations suggested by the research team

Initial data interpretation led to some suggested modifications of the intervention in preparation for a sufficiently or adequately powered randomized controlled trial. In Table 3, we outline several suggested modifications. The table includes the rationale to drive the recommendation. These modifications include increasing referral pathways to clinical services and additional health promotion programs. The recommendations also include some hypothesized programming informed by Veteran feedback, such as having the CHW and primary care team communicate and including the Veteran’s caregivers in visits. Finally, it includes some recommendations coming directly from Veteran feedback, such as non-judgmental approaches to smoking history. These recommendations were later adjusted to include the CHW feedback and comments.

**Table 3:** Research team recommended intervention adaptations based on results

Proposed modification	Rationale
↑ Content on COPD basics, breathing exercises, and inhaler technique	• Participant-stated preference • CHWs reinforced that participants appreciated more education and knowledge
↑ VA resource connections	• Participants said they appreciated the health programming and would be open to more
↑ Referrals to other health and social programs	• CHWs noted the need to connect to additional resources and social services
Include communication pathways between CHWs and the primary care team	• Most participants stated that connecting the two professionals could be helpful
Dedicated content to emphasize non-judgement of smoking history	• One participant shared that some Veterans may feel shame or exclusion due to past smoking history
Optional inclusion of caregiver/family/spouse	• The literature and CHWs expressed the importance of including care givers • Interviewed participants expressed this is unnecessary, so it should not be required

### Qualitative phase 3: CHW qualitative interviews

The two interviewed CHWs said the program offered high quality education material that was helpful to the participants. They emphasized how the program structure, training, and access to clinicians, staff, and support made their job easier. They had spent most of their CHW career focused on asthma, and they reflected on how COPD had a new level of complexity—“It’s like, there is no reversible…. So for me, it’s just a whole deeper level of care that you have to give yourself, and that you have to see through, no matter what mind state you’re in, right?” They discussed the intervention components; connections to other resources, support, and care; and the experience working with Veterans.

### Intervention components

#### Number and cadence of visits

One CHW noted that the number of visits was higher than their asthma and diabetes programs they facilitate and worked well: “We did like 9 or more visits. Which, to me, it was a shock that people wanted to see you 9 times. I thought it was great, we were able to cover everything and still meet their needs, clients’ needs.”

#### Modality (home, video, phone)

They discussed visit modality—“At the beginning I was very, how is this going to work out? I’m not going to get to meet them in person.” One CHW described how their other programs have shifted to all phone as well, and now it is preferred by the CHW: “Me? I prefer, honestly, phone and video. … for me it works so much better because I have less no-shows, and people don’t feel the pressure of having to prepare for us, they have to be home.” The CHW elaborated that after years of doing home visits, she: “was afraid that [phone and video] might not work out, to build relationships. But you know, it has worked out so great.”

### Care connections

#### Connections to other care and resources

The CHWs recommended more resources to provide to the participant. They suggested providing “incentives,” such as the safer cleaning kits (used in their asthma program to reduce exposure to chemical irritants). They also discussed the need to provide additional social support to address health needs beyond direct medical support, such as employment concerns. A CHW suggested templated letters to provide the client “to advocate for their health,” recalling a client facing perceived “employer oppression issues” due to physical restrictions related to their health.

#### Involvement of caregiver, family, and friends in the program

One CHW discussed the necessary role of a caregiver that took part in the visits:

I remember one client, his wife was amazing. She did all of the appointments, she did all of the follow ups, drove him, encouraged him. …. Encourage exercise. Do it together. Some weight lifting, and not too much. She just knew everything about his health. She was his right arm, and he knew it, and it didn’t deter him, it encouraged him. He needed her.

### Working with veterans

Both CHWs discussed their appreciation for Veterans’ interest in learning about their COPD and their persistence and motivation to improve their health. One CHW would reference how working with Veterans stood out differently in her experiences:

You know, I guess overall I would have to say that I was amazed by the clients. All of them, they knew that they had to take care of themselves, it felt to me like, while I could be supportive, they were doing everything already…. Amazing to see that level of focus and seeking support for their own health. And I don’t think you see that everywhere.

### Health outcomes—participant questionnaire

In pre/post analysis, there was clinically significant improvement in COPD symptoms with a decrease in the CAT (mean change: − 4.0, 95% confidence interval: − 7.5 to − 0.5). Similarly, there were clinically significant improvements in several quality of life domains of the CRQ including the Fatigue (1.1, 0.1 to 2.1) and Emotional (0.8, 0.2 to 1.4) scales. The CRQ Mastery and Dyspnea scales increased in the direction of improvement. Medication adherence (ARMS) went down in the direction of improvement, but the change was not significant. Depression and anxiety (HADS) did not substantially change. Self-reported physical activity (PASE) was lower at follow-up (− 68.1, − 118.0 to − 18.1) (Table 2).

## Discussion

In a pilot intervention to improve COPD management provided by CHWs, 81% of eligible Veterans enrolled in the study. Both participants and CHWs found the intervention acceptable, appropriate, and feasible. Moreover, the intervention had high participant satisfaction and initial results showed improved health-related outcomes. In the setting of pandemic disruption, participants preferred home visits but found video visits worked nearly as well. We noted several facilitators to participant’s health behavior change, including social support provided by CHWs. Participants valued increased understanding of this complex disease. The CHWs were able to teach breathing techniques and proper inhaler use which helped participants to breathe better. Participants and CHWs provided feedback on how to improve similar COPD and CHW interventions for Veterans.

We sought to understand acceptability and feasibility of home, video, and phone visits. Home and video were universally preferred, although some participants noted that phone was equally good. Future interventions can consider tradeoffs between convenience, building rapport and trust, as well as opportunities to assess the home environment. Ultimately, the flexibility of having all three modalities made the intervention resilient even under COVID-19 pandemic restrictions.

There were divergent data about inclusion of caregivers. Participants indicated that they would prefer not to have participation of family and friends, consistent with the literature [51]. Other studies have asked the informal caregivers (e.g., spouse, relative, or friend), and found that [52] caregivers desire to be more engaged with the patient’s health services, needing additional information and support [52]. CHWs further emphasized the important role of caregivers during their visit. Given these findings and attention to participant perceived appropriateness, we suggest making an optional module that includes caregivers when mutually acceptable.

Based on qualitative interviews of participants and CHWs, there are several recommended changes to the intervention (Table 3). For example, one participant discussed feeling guilty about smoking and how “I did that [to] myself.” A study of Veterans receiving lung cancer screening found that guilt and shame of smoking contribute to the inability to quit and decreased self-efficacy, due to emotional and cognitive biases that interfere with health behavior change [53]. The authors recommend tailoring interventions, recognizing that some participants might feel they are not qualified or deserving of the support [53]. Interventions can take proactive, non-judgmental approaches to improve participant perceptions of intervention acceptability and appropriateness. Recruitment materials can emphasize inclusivity of smokers and former smokers. Once enrolled, practitioners can proactively discuss these underlying concerns to meet the participants where they are at. Programs can include both physical and mental health aspects to address the increased anxiety and depression experienced in this participant population.

We investigated implementation outcomes, primarily from the patient perspective. Researchers note the benefits of utilizing implementation science early in the planning and pre-implementation phases of research, particularly in designing interventions towards serving vulnerable populations (e.g., Veterans) to improve health equity [54]. To further accelerate health equity, early studies can center on those most impacted, typically the patient [54–56]. However, many implementation science tools and practices are not designed for use with patient-participants and the feasibility of implementing programs into clinical practice is not typically informed by patient-participants [31]. However, home-based interventions (physical and virtual) make patient-participants the owners of the implementation setting and an essential voice in evaluating its feasibility.

Some implementation frameworks have evolved to be more inclusive of the patient-participant in their more current versions [57]. The TDF used in this study is well suited for inter-personal behavioral interventions between patients and their health care professionals [30]. By using the TDF, the results are contextualized in behavior change mechanism and theory, which can advance the generalizability and applicability of the recommended intervention adaptations in Table 3.

Finally, the pilot showed successful recruitment practice and interest, suggesting that these recruitment practices at the VA will make a sufficiently or adequately powered intervention trial possible. We considered several other improvements to address recruitment limitations in a future trail. Many participants screened out due to inclusion criteria to include those with highest-risk COPD, defined as having ≥ 1 exacerbation requiring prednisone. A modified approach based on an accepted guideline of COPD Assessment Test (CAT) score ≥ 10 for treatment eligibility would broaden reach and benefit more high-risk participants. We also screened out participants without a computer or video-enabled device. If fully funded, we anticipate a future research trial may be more successful by providing loaned video-enabled devices and overcoming staffing and COVID-19 pandemic barriers faced in this pilot. This study was provided by unfunded, volunteer staff as part of a training and professional development opportunity. The COVID-19 pandemic began halfway through the recruitment period, potentially impacting enrollment despite the strengths in versatility to work with participants remotely.

We acknowledge several limitations in this exploratory analysis. First, the data sample was small, with loss to follow-up. Those participants that completed data collection may not represent the experiences of all intervention participants more broadly. Second, the pilot required internet connectivity for use of video. The VA has services to help address digital inequities that can limit this bias if brought to full randomized trial. Third, these data offer limited generalizability based on health care system, geography, and disease-focus. Fourth, the overlap with the COVID-19 pandemic restrictions likely influenced some results. Preferences around intervention modality (home, video, telephone), decreased physical activity scores at follow-up, and the social support findings may have been impacted by COVID-19-related isolation. Finally, we did not pre-specify or evaluate specific progression or success criteria. Several recent studies document these best practices to help more objectively and systematically evaluate criteria for recommending moving forward from pilot to full trial [58, 59].

## Conclusion

In summary, our study presents a promising intervention to improve COPD self-management provided by CHWs. These initial results suggest the intervention is acceptable, feasible, and appropriate and could improve health outcomes, including quality of life. Future directions include a sufficiently or adequately powered randomized controlled trial to further test the intervention, with modifications informed by pilot participant and CHW insights to better serve Veterans enrolled in VA health care.

## Supplementary Information

Additional file 1.

Additional file 2.

Additional file 3.

Additional file 4.

## Data Availability

Final de-identified, anonymized datasets analyzed in this study may be shared if requested under a written agreement that adheres to applicable informed consent provisions. Due to ethical restrictions, we are unable to share qualitative data publicly because the data contains potentially identifying and/or sensitive patient information. **Declarations** The views expressed in this article are those of the authors and do not necessarily reflect the position or policy of the Department of Veterans Affairs or the United States Government. This material is the result of work supported by resources from the VA Puget Sound Health Care System, Seattle, Washington.
