# Supplementary material for: Community health workers serving Veterans with chronic obstructive pulmonary disease: a pilot intervention development and feasibility study
Source: Pilot Feasibility Stud. 2026 Jan 3;12:18. doi: 10.1186/s40814-025-01711-8 (PMC12866377; doi:10.1186/s40814-025-01711-8)
Supplement: Supplementary file 2 — Additional file 2. [file 40814_2025_1711_MOESM2_ESM.docx]

Additional File 2:

Participant and CHW Interview Guides

Participant Interview Guide

Interviewer Name:

Date:

Time Start:

Time End:

Hello [Mr./Ms. interview participant name],

My name is [interviewer name]. We are interviewing veterans who participated in the COPD, Community Health Worker program in order to get information that will help us to improve the quality of care we provide.

Your participation will remain confidential. We won’t identify you as a participant in any of our reports. Your participation in this interview is voluntary.

The interview will last 30-45 minutes. You can stop the interview at any time, and let me know if you’d rather not answer a particular question.

Do you have any questions?

In order to make sure we capture all of the information you give us, we would like to record this call. The audio-file for the recording will be stored directly to restricted access file on the VA secure server. Is this okay with you? [Hit record button.] Okay, to confirm, I’m starting the recording. Is this ok with you?

[Generic prompts: If responses are limited or require clarification, probes may be used to elicit more detailed responses. Probes should use words or phrases presented by the participant using one of the following formats:

1. What do you mean by ___________?
2. Can you tell me more about ___________?
3. Can you give me an example of ____________?
4. Can you tell me about a time when ____________?
5. Did you talk about ___________ with your Community Health Worker [or Name]?

1. **Tell me about your experience participating in the COPD-Community Health Worker program.**

*Follow-up with generic prompts in order to elicit rich & detailed description.*

[IF NEEDED]

- 1. What was the most useful part of the program for you?
  2. What was the least useful part of the program for you?
  3. Tell me about having a health coach
     1. What characteristics, qualities, or experiences would be important in a community health worker?
  4. Tell me about the health education part of the program.
     1. Was the book useful for you?

[as NEEDED]

What, if anything, do you like about these materials?

What, if anything, do you not like about these materials?

- - 1. Was there anything else that you wished the program to covered?
  1. Did the Community Health Worker [or Name] connect you to new resources or programs?

[AS NEEDED]

1. Did they connect you to community resources or programs?

ii. Did they connect you to VA or clinical resources or programs?

General probe for questions on meeting modality:

How long did _______ last?

1. Tell me about the home visits?

[AS NEEDED]

- 1. Was there anything that made the home visits easy?
  2. Was there anything that made the home visits difficult?

1. Tell me about having phone visits?

[AS NEEDED]

- 1. Was there anything that made the phone visits easy?
  2. Was there anything that made the phone visits difficult?

1. Tell me about having video visits?

[AS NEEDED]

- 1. Was there anything that made the video visits easy?
  2. Was there anything that made the video visits difficult?

1. Do you prefer home, phone, or video visits?
2. Were there enough visits?
   1. Tell me about the timing between visits.
3. Was setting goals part of the COPD-Community Health Worker program?
   1. Tell me about goal setting.
4. Did you and your primary care team discuss your participation in the COPD, Community Health Worker program?
5. Would it be helpful if your primary care team and the Community Health Worker to communicate with each other?
   1. if no, why not?
   2. If yes, what information would be helpful for them to share with each other?
6. Are there ways in which you think Veterans would want to have family or friends involved in this program?
7. Would you recommend this program for other Veterans with COPD?
   1. Tell me more about that
   2. (if yes) What did you like about the program?
   3. What might the VA consider before offering a Community Health Worker program to more Veterans?
8. Do you get what you need for your COPD at the VA?
   1. What do you need for your COPD that you do not get at the VA?
9. Other than the COPD-Community Health Worker program, have you participated in any education or rehab programs to improve or address your breathing?
   1. IF YES Tell us about those programs?
10. In thinking about the COPD-Community Health Worker program again, how is it similar or different from your other experiences managing your COPD?
11. Do you have any questions for us, or is there anything else you would like to add?

Thank you for participating in this interview.

Appendix C: CHW Interview Guide

Community Health Worker Interview Guide

Interviewer Name:

Date:

Time Start:

Time End:

Hello [Mr./Ms. interview participant name],

My name is [interviewer name]. We are interviewing Community Health Workers who participated in the COPD-Community Health Worker program in order to get information that will help us to improve the quality of care we provide.

Your participation will remain confidential. We won’t identify you as a participant in any of our reports. Your participation in this interview is voluntary.

The interview will last about 1 hour. You can stop the interview at any time, and let me know if you’d rather not answer a particular question.

Do you have any questions?

In order to make sure we capture all of the information you give us, we would like to record this call. The audio-file for the recording will be stored directly to restricted access file on the VA secure server. Is this okay with you? [Hit record button.] Okay, to confirm, I’m starting the recording. Is this ok with you?

[Generic prompts: If responses are limited or require clarification, probes may be used to elicit more detailed responses. Probes should use words or phrases presented by the participant using one of the following formats:

1. What do you mean by ___________?
2. Can you tell me more about ___________?
3. Can you give me an example of ____________?
4. Can you tell me about a time when ____________?

1. If you are comfortable sharing, please tell me about your background and experiences and why you work as a Community Health Worker.
2. How do you build trust in your role as a Community Health Worker? [Interview probe as needed: Think about a client who has been harder to reach or less willing to seek healthcare. How do you let them know that you want to help?]
3. What were the main health concerns of the Veterans that you work with?
4. What Community Health Worker skill, experience, or expertise is needed to better address current concerns of the Veteran community?
5. How do you think participants most benefitted from the program?

1. What elements of the program, if any, best helped participants improve their lung condition?

1. What aspects of Community Health Worker programming do you think participants most responded to?
2. What elements were missing from the program, if any, that could better assist participants to manage their health?

1. Tell me about video visits.

[AS NEEDED]

1. Was there anything that made the video visits easy?
2. Was there anything that made the video visits difficult?
3. Did you prefer home, phone, or video visits?
4. How was building rapport with participants similar or different in person vs. only over the phone?
5. Did you ever discuss with the Veteran’s primary care team about their Veteran’s participation in the COPD-CHW study?
6. What information would be helpful for the CHW and primary care team to share with each other?

1. What might the VA consider before offering the COPD-Community Health Worker program to more Veterans?

1. Other than the COPD-Community Health Worker program, have you worked as a CHW in any other programs to help people manage their chronic diseases?
2. (If yes) Tell me more about that.

1. In thinking about the COPD-CHW program again, how is it similar or different from your other experiences working with patients managing health concerns other than COPD?
2. Did you feel that you were equipped to do your job well?
3. What support was helpful during your training and time working as a community health worker?
4. Describe the operational setting or culture of the COPD-Community Health Worker program or research study.
5. We are going to propose a new study based on this intervention. In response to patient feedback, we are planning to make a few changes to the program. Please let us know your thoughts on the following changes:
   1. Conduct by video and phone only.
   2. Physical activity will be discussed and encouraged, however will not be a mandatory.
   3. Have the CHW connect Veterans to existing VA resources that could help them manage their COPD such as smoking cessation, weigh management, and mindfulness programs.
6. Do you have any questions for us, or is there anything else you would like to add?

Thank you for participating in this interview.
