# Supplementary material for: Community health workers serving Veterans with chronic obstructive pulmonary disease: a pilot intervention development and feasibility study
Source: Pilot Feasibility Stud. 2026 Jan 3;12:18. doi: 10.1186/s40814-025-01711-8 (PMC12866377; doi:10.1186/s40814-025-01711-8)
Supplement: Supplementary file 3 — Additional file 3. [file 40814_2025_1711_MOESM3_ESM.pdf]

TIDieR

Chronic Obstructive Pulmonary Disease - Community Health Worker (COPD-CHW) Home Visits study

**Why:**	From the Background Section: Community health worker (CHW) programs that address are effective, with increasing evidence for improving participant health through disease self-management education and behavior change support. Few or no studies of CHW programs addressing COPD
**What (material):**	From the Methods Section: We utilized materials from the COPD-Self-Management Activation Research Trial (COPD-SMART) intervention, which was a 9-month intervention with COPD education and 25 physical activity sessions. The participant received the COPD-SMART manuals to use for visits, but the CHWs only covered the introductory materials focused on COPD self-management (6 sessions) and developing a physical activity plan (3 sessions).
**What (procedures):**	From the Methods Section: Veteran participants were scheduled for 9 CHW visits over 12 weeks. As part of the first visits, the CHW performed a needs assessment that included a checklist-based home environment review, which could be done by video or phone when home visits were not possible during the COVID-19 pandemic period. The CHW learned about the Veteran's priorities for their COPD, which they incorporated into a COPD management plan. Using motivational interviewing techniques, the CHW discussed any challenges that might exist to implementing the plan and approaches to address those concerns. The CHW worked with the Veteran to set a schedule to cover the 9 visits on a weekly basis. The COPD self-management sessions included: a) understanding COPD and its impact; b) communicating with health care providers; c) understanding medications; d) non-pharmacologic strategies for controlling symptoms; e) exacerbation action plans; f) enhancing physical activity; g) eating healthy; h) smoking cessation; and i) mood. To enhance the section on understanding medications, the CHW trained the Veteran on proper use of an inhaler, using the Teach-to-Goal method. The final three sessions covered motivation for physical activity: getting ready for healthy behavior change, and making a plan to overcome barriers and achieve benefits.
**Who provided:**	From the Methods Section: Three CHWs participated in the pilot intervention. The CHWs worked at the local public health department, with prior work in a clinical program focused on asthma and experience participating in clinical trials. The CHWs serve the greater King County area, and recruitment was limited to this region. They are not Veterans themselves, but two have Veterans in their families. They received 40 hours of training through sessions with the study clinicians (VF and DC) and online courses through the American Lung Association. They met routinely with the study staff and principal investigator (VF) to review all visits and had access to on-call support if needed. They also received technical assistance for video conferencing with study staff.
**How (mode of delivery; individual or group):**	From the Methods Section: The COPD-CHW Home Visits study was initially designed to be delivered through a combination of home and virtual visits (video or phone). Participants were offered additional phone calls between visits to check-in on their COPD self-management goals and confirm their upcoming appointments.
**Where:**	From the Methods Section: Per patient preference, CHW visits were conducted either through video conference technology (VA Video Connect) or by phone, with the exception of the first two participants that were offered home visits before the onset of the COVID-19 pandemic.
**When and how much:**	From the Methods Section: The CHW worked with the Veteran to set a schedule to cover the 9 visits on a weekly basis.

Tailoring: Chronic Obstructive Pulmonary Disease - Community Health Worker (COPD-CHW) Home Visits study

From the Methods Section: The CHW learned about the Veteran's priorities for their COPD, which they incorporated into a COPD management plan.

How well (planned): From the Results Section: We successfully enrolled nine out of the eleven eligible participants (81%), and 100% completed the nine planned study visits.
