# Supplementary material for: Community health workers serving Veterans with chronic obstructive pulmonary disease: a pilot intervention development and feasibility study
Source: Pilot Feasibility Stud. 2026 Jan 3;12:18. doi: 10.1186/s40814-025-01711-8 (PMC12866377; doi:10.1186/s40814-025-01711-8)
Supplement: Supplementary file 4 — Additional file 4. [file 40814_2025_1711_MOESM4_ESM.docx]

Additional File 4:

| **Overview of measurements performed** | | | |  |
| --- | --- | --- | --- | --- |
|  | **Baseline** | | **4-months** | |
| **Data Collection** | Surveys  (mail) | TTG scores  (CHW Visit #1) | Surveys  (mail) | Video visit by research staff |
| Consent and randomization | **🗸** |  |  |  |
| CRQ | **🗸** |  | **🗸** |  |
| Physical activity (PASE) | **🗸** |  | **🗸** |  |
| Inhaler technique (TTG scores) |  | **🗸** |  | **🗸** |
| Cognition (MiniCog) |  | **🗸** |  |  |
| Home environment questionnaire |  | **🗸** |  |  |
| Medication adherence (ARMS) | **🗸** |  | **🗸** |  |
| Psychological symptoms (HADS) | **🗸** |  | **🗸** |  |
| Health care utilization | **🗸** |  | **🗸** |  |
| Social Support (MOS-SSS) | **🗸** |  | **🗸** |  |
| Readiness to change for PA | **🗸** |  | **🗸** |  |
| Clinical characteristics |  |  |  |  |
| Demographics | **🗸** |  |  |  |
| Smoking status | **🗸** |  | **🗸** |  |
| Alcohol use (Audit C) | **🗸** |  |  |  |
| General health (SF-36) | **🗸** |  | **🗸** |  |
| Physical activity checklist | **🗸** |  |  |  |
| Belief in medications (BMQ) | **🗸** |  |  |  |
| Social Support (MOS-SS) | **🗸** |  |  |  |
| COPD severity scale | **🗸** |  | **🗸** |  |
| COPD Assessment Test (CAT) | **🗸** |  | **🗸** |  |
| Acceptability (AIM) |  |  | **🗸** |  |
| Appropriateness (IAM) |  |  | **🗸** |  |
| Feasibility (FIM) |  |  | **🗸** |  |
| Satisfaction with technology |  |  | **🗸** |  |
| Satisfaction with Program |  |  | **🗸** |  |
| Spirometry* |  |  |  |  |
| CHW=Community Health Worker; CRQ=Chronic Respiratory Questionnaire; SF-36=Short Form-36; PASE=Physical Activity Scale for the Elderly; TTG=Teach-to-Goal; ARMS=Adherence to Refill and Medications Scale); BMQ=Beliefs about Medicines Questionnaire; MOS-SSS=Medical Outcomes Study-Social Support Survey; PA=physical activity; AIM=Acceptability of Intervention Measure; IAM=Intervention Appropriateness Measure; FIM=Feasibility of Intervention Measure  * Spirometry is not required, and will be abstracted from the EMR | | | | |
